# Food with Extended Shelf Life Featuring Ingredients Derived from Fruits, Vegetables, and Wild Edible Plants: Nutritional, Functional, and Sensory Properties

**DOI:** 10.3390/foods15111886

**Published:** 2026-05-27

**Authors:** Nebojša Đ. Pantelić, Vesna V. Antić

**Affiliations:** Department of Chemistry and Biochemistry, Faculty of Agriculture, University of Belgrade, Nemanjina 6, 11080 Belgrade, Serbia

## 1. Introduction

The growing demand for safe, high-quality, and minimally processed foods has intensified research efforts toward the development of products with extended shelf life while maintaining their nutritional and sensory attributes. Shelf-life extension is a critical aspect of modern food systems, as it directly impacts food safety, economic efficiency, and the reduction in food waste, which remains a global challenge [1,2]. In this context, natural ingredients derived from fruits, vegetables, and wild edible plants have gained increasing attention due to their potential to improve food stability while simultaneously enhancing functional and nutritional properties [3].

Fruits and vegetables are rich sources of bioactive compounds, including polyphenols, carotenoids, vitamins, and dietary fibers, which have been associated with antioxidant, antimicrobial, and health-promoting effects [4,5]. These compounds can contribute not only to the preservation of food products by inhibiting oxidative and microbial degradation processes, but also to the development of functional foods with added health benefits. In recent years, wild edible plants have also emerged as valuable resources, offering a diverse range of phytochemicals with unique biological activities and potential applications in food preservation and formulation [6].

The incorporation of plant-derived ingredients into food systems has been explored through various technological approaches, including the use of extracts, powders, essential oils, and encapsulated bioactive compounds. Such strategies aim to improve product stability and shelf life without relying on synthetic additives, aligning with current consumer preferences for clean-label products [7]. Moreover, the impact of these ingredients on sensory characteristics, such as taste, aroma, texture, and color, is of particular importance, as consumer acceptance remains a key factor in the successful development of novel food products.

Recent studies have demonstrated that the application of natural plant-based ingredients can significantly enhance both the functional and preservation properties of food products, while maintaining or even improving their sensory quality [8,9]. For instance, the use of fruit- and plant-derived bioactives has been shown to influence oxidative stability, microbial growth, and overall product quality during storage, highlighting their potential as sustainable alternatives to conventional additives. In this regard, comprehensive evaluations that integrate nutritional, functional, and sensory aspects are essential for the development of food products with extended shelf life and improved overall quality [10].

The present Special Issue aims to provide an overview of recent advances in the use of ingredients derived from fruits, vegetables, and wild edible plants for the development of foods with extended shelf life. The contributions collected in this issue address various aspects of this topic, including the characterization of bioactive compounds, their functional properties, and their impact on food quality and stability. Together, these studies highlight the growing importance of natural ingredients in the design of innovative and sustainable food systems.

## 2. An Overview of Published Articles

The articles published in this Special Issue reflect current research trends related to the development of food products with extended shelf life through the incorporation of ingredients derived from fruits, vegetables, and wild edible plants. The published contributions address various aspects of food science, including the characterization of bioactive compounds, the evaluation of functional and antioxidant properties, and the impact of plant-derived ingredients on the stability, preservation, and sensory quality of food products. Particular attention is also given to the sustainable valorization of plant materials and food by-products as functional ingredients in innovative food formulations. Collectively, these studies demonstrate the growing importance of natural and sustainable approaches in the formulation of functional foods and clean-label products with improved quality and consumer acceptance. In the following section, a brief overview of the articles published in this Special Issue is presented.

Mitrevski et al. (contribution 1) investigated the potential application of beetroot powder as a functional ingredient in the formulation of low glycemic index biscuits enriched with betaine and mineral nutrients. The study demonstrated that the incorporation of beetroot powder improved the nutritional profile of the biscuits by increasing the content of bioactive compounds and minerals, while also contributing to reduced acrylamide formation and acceptable sensory properties during storage. Furthermore, the developed biscuits exhibited a low glycemic index, highlighting their potential as functional confectionery products with improved health-related characteristics.

Pascariu et al. (contribution 2) investigated the incorporation of elderberry extract into yogurt formulations in order to enhance antioxidant capacity and improve microbiological stability during refrigerated storage. The study demonstrated that the addition of elderberry extract significantly increased the total phenolic and flavonoid contents, enhanced antioxidant activity, and maintained the viability of beneficial lactic acid bacteria over a 21-day storage period. Furthermore, the enriched yogurts exhibited improved sensory acceptance and showed potential as functional dairy products formulated with natural bioactive ingredients and reduced reliance on synthetic preservatives.

Sáez et al. (contribution 3) evaluated the application of white grape marc extract as a natural preservative for refrigerated gilthead seabream filets in order to extend shelf life and improve product quality. The study demonstrated that grape marc extract significantly reduced lipid oxidation and psychrophilic bacterial growth during 14 days of cold storage, while also preserving firmness and improving several color parameters compared to untreated samples and, in some cases, even outperforming ascorbic acid treatment. The authors concluded that grape marc extract represents a promising natural alternative to synthetic fish preservatives, while simultaneously contributing to the valorization of winery by-products within a circular bioeconomy approach.

Muhović et al. (contribution 4) investigated whether blending cold-pressed flaxseed oil with apricot kernel, sesame, and black cumin oils could improve its oxidative stability and nutritional quality without thermal processing. The study showed that all blends enhanced the physicochemical properties of flaxseed oil, with apricot kernel oil producing the greatest improvement in oxidative stability, extending the induction period by up to 60%, while black cumin oil most strongly increased antioxidant capacity and phenolic content. The authors concluded that oil blending is an effective natural strategy for stabilizing flaxseed oil and enhancing its functional and nutritional properties, offering potential applications in food and nutraceutical products.

Coyago-Cruz et al. (contribution 5) examined the dynamics of bioactive compounds in the pulp, peel, and seeds of *Salacca zalacca* (“Salak”) during three ripening stages and evaluated their antioxidant and antimicrobial activities. The study found that the peel contained the highest concentrations of phenolic compounds, particularly chlorogenic, caffeic, and ferulic acids, while the pulp showed the highest levels of organic acids and antioxidant activity. Additionally, pulp and peel extracts exhibited antimicrobial effects against *Escherichia coli* and *Staphylococcus aureus*, whereas the seeds showed no antimicrobial activity. The authors concluded that, besides the edible pulp, the peel represents a valuable source of bioactive compounds with potential applications in functional foods and natural antimicrobial products.

Jaramillo-De la Garza et al. (contribution 6) investigated the use of extruded avocado seed flour as a functional ingredient in bread production. The study showed that extrusion and enzyme-assisted wet milling improved the flour’s technological properties, particularly water and oil absorption capacity, while substantially reducing acetogenins and antioxidant compounds associated with bitterness. Bread formulations containing 5% extruded avocado seed flour maintained consumer acceptability in terms of texture, appearance, and overall liking, while improving aroma compared with control bread. Although enriched breads had lower loaf volume and higher density, the results demonstrated that extruded avocado seed flour can be successfully valorized as a sustainable, fiber-rich ingredient for bakery products, supporting the circular economy and clean-label food development.

## 3. Conclusions

The studies published in this Special Issue highlight the growing interest in the use of ingredients derived from fruits, vegetables, and wild edible plants for the development of food products with improved quality, functionality, and extended shelf life. The presented contributions demonstrated that plant-based ingredients and natural bioactive compounds can enhance antioxidant potential, oxidative and microbiological stability, and sensory characteristics of various food systems, while also supporting the development of clean-label and functional products.

In addition to their technological and nutritional benefits, several studies emphasized the importance of sustainable approaches through the valorization of plant materials and agro-industrial by-products as valuable food ingredients. Collectively, the articles included in this Special Issue provide insight into current advances and future opportunities in the formulation of innovative and sustainable food products that meet both consumer expectations and modern food industry demands.
